# Intersectionality and health-related stigma: insights from experiences of people living with stigmatized health conditions in Indonesia

**DOI:** 10.1186/s12939-020-01318-w

**Published:** 2020-11-11

**Authors:** Sarju Sing Rai, Ruth M. H. Peters, Elena V. Syurina, Irwanto Irwanto, Denise Naniche, Marjolein B. M. Zweekhorst

**Affiliations:** 1 Athena Institute, Faculty of Science, Vrije University Amsterdam, VU Amsterdam, De Boelelaan 1085, 1081 HV Amsterdam, The Netherlands; 2 Barcelona Institute for Global Health (ISGlobal), University of Barcelona, Barcelona, Spain; 3 Faculty of Psychology, Atma Jaya Catholic University, Jakarta, Indonesia

**Keywords:** Health-related stigma, Multiple stigma, Intersectionality, Indonesia, HIV, Leprosy, Schizophrenia, Diabetes

## Abstract

**Background:**

Health-related stigma is a complex phenomenon, the experience of which intersects with those of other adversities arising from a diversity of social inequalities and oppressive identities like gender, sexuality, and poverty – a concept called “intersectionality”. Understanding this intersectionality between health-related stigma and other forms of social marginalization can provide a fuller and more comprehensive picture of stigma associated with health conditions. The main objective of this paper is to build upon the concept of intersectionality in health-related stigma by exploring the convergence of experiences of stigma and other adversities across the intersections of health and other forms of social oppressions among people living with stigmatized health conditions in Indonesia.

**Methods:**

This qualitative study interviewed 40 people affected by either of four stigmatizing health conditions (HIV, leprosy, schizophrenia, and diabetes) in Jakarta and West Java, Indonesia between March and June 2018. Data was analyzed thematically using an integrative inductive-deductive framework approach.

**Results:**

The main intersectional inequalities identified by the participants were gender and socioeconomic status (*n* = 21), followed by religion (*n* = 13), age (*n* = 11), co-morbidity (*n* = 9), disability (*n* = 6), and sexuality (*n* = 4). Based on these inequalities/identities, the participants reported of experiencing oppression because of prevailing social norms, systems, and policies (macro-level), exclusion and discrimination from societal actors (meso-level), and self-shame and stigma (micro-level). While religion and age posed adversities that negatively affected participants in macro and meso levels, they helped mitigate the negative experiences of stigma in micro level by improving self-acceptance and self-confidence.

**Conclusion:**

This study uncovered how the experience of health-related stigma intersects with other oppressions originating from the various social inequalities in an individual’s life. The findings highlight the importance of acknowledging and understanding the multi-dimensional aspect of lives of people living with stigmatized health conditions, and warrant integrated multi-level and cross-cutting stigma reduction interventions to address the intersectional oppressions they experience.

**Supplementary Information:**

The online version contains supplementary material available at 10.1186/s12939-020-01318-w.

## Background

Health-related stigma is a complex phenomenon rooted in social inequality, power asymmetry, and systemic hierarchy that mediate the process of stigmatization by othering and oppressing those affected [1–3]. Such experiences of discrimination, oppression, and marginalization - enacted and reinforced by systems, institutions, and social actors - often have negative social, psychological, behavioral, and medical effects on people living with stigmatized health conditions [1, 4–6]. Further adding to the complexity, studies have found that health-related stigma does not exist in isolation, but actually interacts and intersects with other forms of social marginalization and oppression to create a compounding experience of stigma which negatively impacts those affected [4, 7–9].

With emerging studies, researchers have found linkages of stigma associated with health conditions to other forms of marginalization due to race, gender, ethnicity, socioeconomic status, sexual orientation, age, etc. Studies have reported on such intersections of health-related stigma with other forms of social oppression and inequities using different terms like “double stigma” [10, 11] or “multiple stigmas” [12, 13] depending on the number of social categories explored, or as “intensified stigma” [14, 15] to describe the compounded experience of such oppressions. Such intersectional experience of stigma associated with a person’s health condition and adversities related to oppressive social identity/inequalities like gender, sexuality, and poverty can lead to concealment of the condition, social exclusion and isolation, and hamper access to health services, employment, and education [1, 4–6, 8, 16]. These evidences of intersectional relationship between health-related stigma and other forms of social marginalization indicate that stigma related to health conditions is not only a public health issue, but also a social justice issue [9, 17]. Further, it also shows prospects and value of embedding the concept of intersectionality into the theory of health-related stigma to elaborate and improve on the understanding of stigma associated with health conditions.

The concept of intersectionality was first introduced by Kimberlé Crenshaw in order to highlight the dynamics between race and gender, and the overlapping oppressions that African American women face as a result of such an intersection [18]. Intersectionality, as a theory, acknowledges the complexity and multidimensionality of people’s lives, and posits that the social oppression they may experience actually originate from an intersection of different social inequalities and oppressive identities rather than from a singular marginalized identity [18, 19]. The theory also acknowledges how such intersectional dynamics between different social inequalities and identities are contextual and may change over time and be different in different cultural and geographical settings [18–20]. Intersectionality thus has much to offer to the field of health-related stigma in providing an improved understanding of the dynamic interaction and interplay of different social oppressions and marginalized identities and how they shape the experience of stigma among people living with health conditions.

This paper seeks to improve the conceptualization of health-related stigma by employing the paradigm of intersectionality to elucidate the relationships and dynamics between health-related stigma and other adversities arising from social oppressions and identities. Further, this paper focuses on exploring such intersectional experiences across four different health conditions – two infectious (HIV and leprosy), and two non-communicable conditions (a mental illness – schizophrenia, and a chronic metabolic disorder - diabetes) in Indonesia. The unique position of Indonesia as a lower-middle income country (LMIC) undergoing an epidemiological transition with higher burden of both infectious and non-communicable diseases [21–25], combined with its rich cultural and historical context [26, 27] provides an ideal setting for exploration of the convergence of experiences of stigma across the intersections of health and other forms of social inequality (age, gender, ethnicity, religion, poverty) among people living with health conditions.

### The context of Indonesia

Indonesia is the world’s fourth most populated nation with a population of over 267 million [28]. It has both rich and diverse culture with over 633 recognized ethnic groups and 300 native languages scattered over 17,000 islands [26]. It is also a nation with the highest population of Muslims, while the country is pluralist with six officially recognized religions [26, 28]. As with many other LMICs undergoing the process of development and socio-economic transition, Indonesia also faces several problems. Around 25.1 million Indonesians live below the poverty line (20.6% population) [28]. This has resounding effect in the health and quality of life of Individuals [29]. There are also issues of gender inequality and persecution of sexual minorities that are rooted in socio-cultural and religious conservatism. The issue of gender inequality is evident from Indonesia’s ranking (103 out of 162 countries) in the 2018 Gender Inequality Index [30].

Indonesia, like most LMICs, also has high burden of both communicable and non-communicable diseases. It has the highest prevalence of HIV in southeast Asia (0.4%) with 640,000 people living with HIV in 2018 [31]. It also ranks third in the world in terms of prevalence of leprosy [32]. It also has high prevalence of non-communicable health conditions like diabetes mellitus (6.7% in adults) [33] and schizophrenia (around 1%) [34]. Besides the high burden, these four health conditions are also stigmatized in the Indonesian society because of the prevalent social norms, and associated negative stereotypes and misinformation [35–39].

### Multi-level conceptualization of Intersectionality and health-related stigma

Intersectionality and health-related stigma are both conceptualized in multi-levels of influence, which complement each other. Both theories often categorize the experiences into three levels – macro, meso and micro levels [8, 20, 40]. The macro level, which is also called structural level, deals with policies, practices, norms, power structures, and dynamics that restrict, undermine, subjugate, or oppress the marginalized. The meso or interpersonal level deals with interactions with societal actors that give way to perpetration of prejudice and discrimination towards those oppressed based on the prevalent negative stereotypes in the society. Finally, the micro level, which is also called intrapersonal or individual level, is where the experiences from macro and meso levels give rise to negative internalized feelings among those affected [19, 40–42]. This complementarity of conceptualization of the two concepts presents the potential to inform the complex interplay of social oppressions and associated adversities with health-related stigma to explain their underpinning process of operation.

This multi-level conceptualization has been used in previous studies by Logie et al. [8] and Watkins-Hayes [43] to investigate the intersectionality of HIV-related stigma with other stigmas and adversities based on varied social inequalities like race, gender, sexual orientation, etc. This paper advances the use of this conceptualization beyond HIV, and uses this multi-level framework to explore the intersectional experience of health-related stigma across four different health conditions and other social adversities at the individual (micro) level, derived from the interactions at the interpersonal (meso) and structural (macro) levels.

## Methods

This paper draws on the qualitative empirical research conducted in in Jakarta and West Java in Indonesia between March and June 2018 among people living with four different stigmatized health conditions, viz. HIV, leprosy, schizophrenia, and diabetes. These four conditions were purposively chosen because of their diverse nature and etiology (infectious vs non-communicable), and causes of stigmatization in the Indonesian society [36–39, 44].

This exploratory study applies a critical and constructivist perspective to explore and analyze the construction of intersectional experiences of health-related stigma [45, 46]. The perspective applied in this study looks at the interactions, interrelations, and interplay between the intersectional experiences of individuals within a shared socio-cultural context, with which we analyze, make sense of, assimilate and report the findings.

### Study population and selection

The study locations were chosen based on the higher proportion of individuals with different health conditions in those regions. Participants with HIV and diabetes were recruited in Jakarta, those affected by leprosy in Cirebon, West Java, and those with schizophrenia in Jakarta and Cianjur, West Java. Purposive convenience sampling was used to recruit participants from the community through referrals and recruitment by community-based organizations and peer-support groups related to the four different conditions. Those who were over the age of 16 (age of consent) and who were willing and consented to participate were included in the study. In total 40 participants (ten per health condition) were recruited and interviewed.

### Data collection

The interview guide was developed based on the multi-level conceptualization of intersectionality and health-related stigma and validated through consultations with co-authors (RMHP, EVS, MBMZ, I), other research experts, and representatives from non-governmental organizations and the different disease groups (a list of stakeholders involved in the consultation process is presented in Supplementary file 1). The interview guide included open questions on experience of living with a stigmatized health condition, the existence of other adversities and inequalities in the participant’s life, the experiences of stigmas as a result of the existing adversities and inequalities. Pilot interviews were first conducted with four participants (one each from each health condition) in order to test the applicability and appropriateness of the interview guide and the interview technique. The interview guide was subsequently refined and adjusted upon piloting.

Special precautions were taken to protect the privacy of the participants during the interviews so as to avoid further stigmatization. For the participants who reported of having a publicly disclosed health status and being open and comfortable in their personal setting, interviews were held in their homes. For others, the interviews were carried out at the non-governmental organization offices in privacy. The interviews started in an exploratory manner asking about the general daily experience of living with their health condition and progressed towards more in-depth questions on specific examples of personal experiences related to their health condition and other social inequities and oppressive identity, and their impact, and lasted for an average of 1 hour. In-depth probing was conducted until saturation of response. All the data was collected by a team of three Indonesian research assistants under the supervision of the main researcher (SSR). The research team was trained on interview techniques and strategies for qualitative data collection prior to fieldwork (in the first week of March). Data was recorded electronically, transcribed verbatim, translated, managed, and analyzed with the qualitative software package ATLAS.ti (version 8.4.18).

### Data analysis

Data analysis was done by SSR, with supervision of EVS and RMHP, using an integrative inductive deductive framework approach [47]. This included coding the data both inductively (data driven) and deductively (based on the multi-level theory used in the interview guide), categorizing data under common themes. A coding framework was developed using codes derived from the data (inductive) and the multi-level of influence model of intersectionality and health-related stigma (deductive) [8, 19, 40–42]. The codes were then categorized based on the emerging themes into the following intersectional stigmas: gender, poverty, religion, age, comorbidity, disability, and sexuality. Each thematic category was further sub-categorized into macro, meso and micro levels. An outline of the final categories and sub-categories is presented in Supplementary file 2.

### Validity

The analytical process was peer debriefed and discussed in each stage to ensure the quality and validity of codes, categories, and themes. The authors involved in the data analyses made sure all codes, categories, and themes were obtained until the point of saturation. The results of the analysis were carefully reviewed and refined in all stages. Rigorous vetting and deliberation were carried out among the authors in determining the relationships between the categories and developing themes. The final themes were thus obtained after consensus from all authors on the final themes and the logical connection and pathway they describe.

Reflection on the positionality of researchers is essential in appraising the effect of their knowledge, culture, perception, and interests on the understanding of the social phenomena being studied [48]. In this study an interdisciplinary research team was involved which consisted of researchers from different scientific disciplines (epidemiology, global/public health, sociology, psychology, disability studies, development studies, and health communication, pharmacy) and countries (Indonesia, Netherlands, Nepal, Russia, Spain). Most team members have more than 10 years of experience with studying health-related stigma in, among others, HIV, leprosy, mental illness, disability and related issues.

## Results

Sociodemographic characteristics of the participants (*n* = 40; ten each with HIV, leprosy, schizophrenia, and diabetes) is presented in Table 1. The mean age of the participants was 40.9 years (SD 11.54; range 19 to 75 years) and the mean duration of living with the health condition was 10.4 years (SD 5.8; range 1 to 25 years). Among the participants, 18 were male (45.0%), 21 female (52.5%), and 1 transgender (2.5%). Most participants belonged to the two main ethnic groups, viz. Java (40%) and Sunda (20%), while the others belonged to the minority ethnic groups: Tionghoa (15%), Batak (7.5%), Ambon (5.0%), and others (12.5%). The majority of the participants were Muslims (62.5%), followed by Christians (32.5%) and Buddhists (5.0%).

**Table 1:** Sociodemographic characteristics of participants

	Total participants = 40			
Participants’ characteristic	***HIV***	***Leprosy***	***Schizophrenia***	***Diabetes***
	***n (%)***	***n (%)***	***n (%)***	***n (%)***
**Age (in years), mean [SD]**	40.4 [7.17]	34.30 [9.64]	35.0 [7.40]	54.0 [10.0]
**Duration of living with the health condition (in years), mean [SD]**	12.1 [4.15]	8.40 [5.06]	10.50 [5.12]	10.9 [8.43]
**Gender**				
***Male***	3 (30%)	4 (40%)	9 (90%)	2 (20%)
***Female***	6 (60%)	6 (60%)	1 (10%)	8 (80%)
***Transgender***	1 (10%)	0 (0%)	0 (0%)	0 (0%)
**Residence**				
***Rural***	1 (10%)	10 (100%)	2 (20%)	0 (0%)
***Urban***	9 (90%)	0 (0%)	8 (80%)	10 (100%)
**Ethnicity**				
***Java/Sunda (majority)***	4 (40%)	10 (100%)	4 (40%)	6 (60%)
***Others (minority)***	6 (60%)	0 (0%)	6 (60%)	4 (40%)
**Religion**				
***Islam***	6 (60%)	10 (100%)	6 (60%)	3 (30%)
***Christianity***	4 (40%)	0 (0%)	4 (40%)	5 (50%)
***Buddhism***	0 (0%)	0 (0%)	0 (0%)	2 (20%)
**Education**				
***> =Senior high***	10 (100%)	4 (40%)	10 (100%)	9 (90%)
***< =Junior high***	0 (0%)	6 (60%)	0 (0%)	1 (10%)
**Occupation**				
***Employed***	10 (100%)	7 (70%)	6 (60%)	6 (60%)
***Unemployed***	0 (0%)	3 (30%)	4 (40%)	4 (40%)
**Income**				
***< =USD140***	0 (0%)	7 (63.6%)	7 (70%)	5 (50%)
***> USD140***	10 (100%)	1 (9.1%)	0 (0%)	3 (30%)
***Not reported***	0 (0%)	2 (18.2%)	3 (30%)	2 (20%)

### Intersectional social inequalities and identities

Except for four respondents with diabetes, the other 36 respondents reported stigma experiences owing to their health condition. The experiences of health-related stigma of the participants is described elsewhere in detail [38]. Participants reported the intersection of stigma associated with their health condition with adversities from other social inequalities/oppressive identities they bear. Gender and socioeconomic status were the most reported social inequalities that intersected with the experiences of health-related stigma by 21 participants. This was followed by religion (*n* = 13) and age (*n* = 11). Those who identified as having a comorbid health condition (*n* = 9), disability (*n* = 6), and being sexual minority (gay and transgender; *n* = 4) also reported stigmas associated with their identity. The main inequalities and/or identities that the participants reported as intersecting with stigma associated with their health conditions are presented in Table 2 below.

**Table 2:** Reported social inequalities and/or identities that intersect with health-related stigma

Participant’s health condition	Reported social inequalities/identities that intersect with health-related stigma						
	Gender	Socioeconomic status	Religion	Age	Comorbidity	Disability	Sexuality
HIV	8	5	4	2	3	0	3
Leprosy	4	4	2	0	1	3	0
Schizophrenia	7	9	7	5	2	3	1
Diabetes	2	3	0	4	3	0	0
**Total**	**21**	**21**	**13**	**11**	**9**	**6**	**4**

*Based on n = 36 participants reporting intersection of health-related stigma with social inequalities/identities*

### Intersectional experiences of stigma and other adversities

**Fig. 1:**
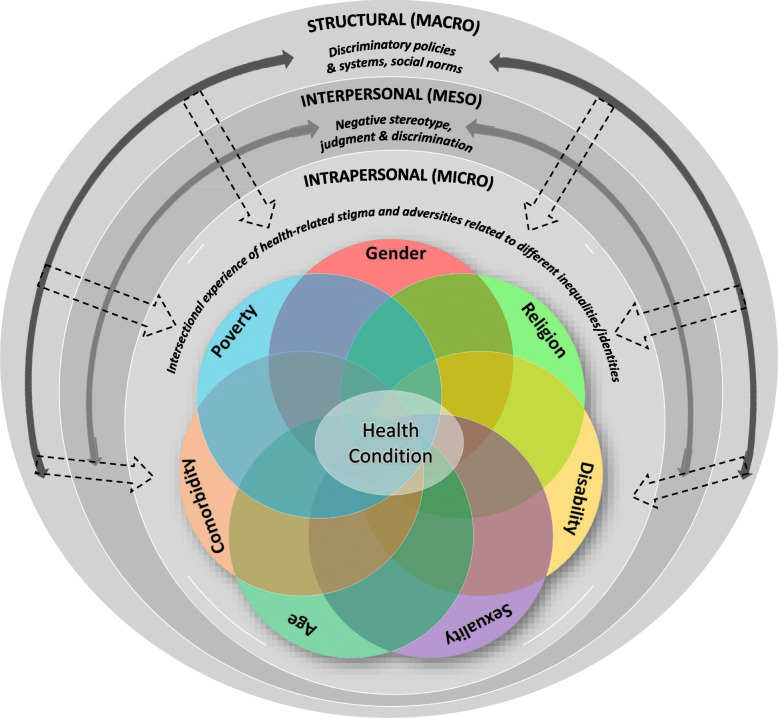
Multi-level conceptualization of intersectionality between health-related stigma and other adversities originating from reported social inequalities and/or identities of individuals living with HIV, leprosy, schizophrenia, and diabetes

Participants reported multi-level intersectional experiences. Figure 1 illustrates the identified intersectional social inequalities/identities and how they intersect with each other and stigma related to an individual’s health condition to ultimately shape the intersectional experience of health-related stigma in the macro, meso, and micro levels. In the macro-level, participants described exclusionary policies and practices in healthcare settings and workplace, and oppressive social norms in the Indonesian society that devalue and discredit people based on the different social inequalities/oppressive identities they hold in conjunction with their health condition. In the meso-level, participants discussed experiences of being stereotyped, judged, and discriminated against by societal actors (healthcare providers, employers and work colleagues, neighbors, and family and friends). In the micro-level, they discussed how the experiences in the macro- and/or meso-level impacted their self-confidence and self-esteem, and shaped their personal experience of stigma owing to their health conditions and the different identities they bear, in the form of internalized shame, guilt, and fear of impending negative judgment and discriminatory behavior from others.

In the following section, we first present the multi-level experience of intersectionality of health-related stigma with a singular inequality or oppressive identity of the participants, then we present four case studies illustrating the interaction and interplay of the different social inequalities/oppressive identities that shape the stigma experiences of the participants.

### Gender: double burden of disease and deviation from gender roles

Participants discussed how perceptions and norms surrounding gender affected their experience of health-related stigma (macro level). Both male and female participants with HIV and leprosy talked about how women are subjugated in the Indonesian society and lack agency. One participant with HIV reported: *“Stigma is more severe for women with HIV. They face double stigma as they are automatically considered to be either promiscuous or have a bad character for having the disease just because they are women…. even if they got infected by their spouse/partner (Male, 44, I36)”.* Among participants with schizophrenia, men were considered to be more stigmatized compared to women. They talked about how the society expects men to be strong, while women are either tolerated or pitied. One participant said: *“A man is expected to be the head of the family, but if he’s schizophrenic he will be a liability to his family and the society because he won’t be able to work. Women, on the other hand, tend to follow their husband’s path – so they are less judged and stigmatized (Male, 32, I40)”.* One participant with HIV who identified herself as a transgender woman talked about how it is even more difficult for transgender individuals as they are, according to her, *“shunned and excluded in every facet of the Indonesian society”* because of their gender identity.

Participants with HIV, leprosy, and diabetes discussed how women are much harshly treated compared to men as a result of prevalent social and gender norms (meso level). One woman with diabetes recounted of being considered irresponsible with people commenting that *“being a woman you should have made better choices”*. Female participants with HIV also talked about how women with HIV are prone to experiencing violence and abuse from their partner and family. In the micro-level, participants described feelings of shame, loneliness, and hopelessness because of their gender and health condition. One transgender participant with HIV talked about feeling lonely and losing hope in life because of both her gender identity and health condition. She said: *“I feel lonely. I live alone. With my sickness, it’s even harder. I feel like if I die - no one will cry for me (Transgender woman, 58, I36)”.* Further in the micro-level experience, female participants with HIV discussed how women are biologically susceptible to negative effects of HIV – from risk of complications in pregnancy, breastfeeding, and mother-to-child transmission of HIV, to having greater likelihood of getting cervical cancer. Participants with diabetes also explored the biological dimension of gender, where most talked about how diabetes affects sexual functioning – particularly in men. One participant said: *“Men feel insecure and ashamed because diabetes affects their sex life and can cause impotence. When people hear that the husband has diabetes, they usually pity his wife (Male, 50, I30)”.*

### Socioeconomic status: intensified stigma of being poor, sick, and no access to healthcare

Participants reported how their socioeconomic status affected their experience of health-related stigma. They particularly talked of poverty as one of the most important intersectional adversities in their lives. Participants indicated two ways in which poverty affected their experience of stigma. The first was in regards to the social standing of individuals in the community (macro level). Participants talked about how poor people bear the *“double burden of stigma of being poor while having a health condition”*, while wealthy people are respected despite their health condition. Participants talked about how society treats people with money with respect, while those who are poor bear the double burden of stigma of being poor while having a health condition (meso level). One participant with leprosy said: *“In my area, those who come from lower income family are disrespected and shamed. If there’s someone from a rich family with leprosy…they are given respect and treated better solely based on his/her family background, and not based on his/her disease (Male, 25, I9).”*

Second, was in regards to their ability to afford medicines and health services (micro level). People with HIV, schizophrenia, and diabetes talked about how it was difficult for them to buy medicines and pay for health services because of their economic condition. The participants argued that proper treatment and management of their disease was the first step towards self-acceptance and aversion of stigma – however, in its absence people felt more stigma. One participant with diabetes noted: *“The most important factor… [that affects stigma] ... is a person’s economic condition. It is very difficult for poor people to accept themselves and their health condition, because they struggle to survive and buy medicines for their treatment. They are also not well informed about their health condition. But for people with good economic condition, they do not have to struggle for survival. They can buy whatever medicines, no matter how expensive, and it’s easier for them to accept their condition. (Female, 52, I30)”.* Participants with schizophrenia talked about how being poor and having a debilitating condition like schizophrenia instigates a vicious cycle of stigma where poor people with schizophrenia cannot work, which further worsens their health condition, economic condition, social standing, and stigma. One participant with schizophrenia said: *“Schizophrenia is usually paralyzing [debilitating]… they [schizophrenics] can’t do any activity… they can’t work. Even though some of them are working, most are paralyzed [debilitated] and stay at home (Male, 37, I37)”.*

While most participants talked about how the Indonesian national health insurance has now made access to medication and health services better through subsidization and free services, they also reported how this has still not improved stigma experiences among poor in the health care setting (macro level). Participants from all four disease groups talked about how they are not able to access treatment services not covered under the insurance which makes it difficult for them to break the vicious cycle of stigma. One participant with schizophrenia specifically talked about how the practice of *“pasung”* (shackling/forced captivity) is still prevalent among people coming from impoverished communities as they cannot afford specialized care and rehabilitation which is not covered by the insurance.

### Religion: duality of perpetuation and mitigation of stigma

Participants described how their religion affected their experience of health-related stigma in their daily lives. People with HIV, leprosy, and schizophrenia talked about the prevalent religious beliefs that their health condition was a result of their sin (macro level). Participants with schizophrenia mostly talked about how mental illnesses like schizophrenia were particularly prone to religious stigmatization on the grounds of sin and religious deviance. One participant with schizophrenia reported: *“People with mental illness are not counted as Moslems. When we pray, our prayer is not considered legitimate in God’s eyes (Male, 39, I20)”.*

Participants also talked about how because of their religious association and health condition, they were stigmatized and *“pushed away”* by people in the community (meso level). One participant with HIV who identified as a Christian said: *“I feel like religion plays a big role [in stigmatization] … people assume that you are the filthiest person on earth, the most sinful. They treat you badly because of it. I think it happens in all religions (Female, 39, I4)”.* Another participant with HIV who identified as a Muslim further elaborated on religious persecution that people with HIV face: *“Sometimes when other Muslims find out about someone that has HIV, they see them badly. That is why we can’t go anywhere freely. […] I don’t know about other religions, but in my religion, there is still intolerance and antipathy [towards people with HIV] (Male, 44, I36)”.*

Participants discussed how because of the prevailing religious beliefs, they prefer to not disclose their condition in front of others to avoid stigmatization (micro level). One participant with schizophrenia talked about how the religious misconception exists in the society and that he also accepts it: *“Some of them become cautious towards me because from a religious perspective mental illness are related to bad spirits. I also understand them and what they were thinking…. because sometimes I experience a phase when I can’t differentiate between good and bad behavior (Female, 37, I21)”.*

However, while religious norms and beliefs perpetuated stigmatization of people with health conditions, the participants also reported how their religious identity helped them overcome stigma through faith and self-acceptance (micro level). Regardless of their religious affiliation, participants with HIV (*n* = 3), leprosy (*n* = 5), schizophrenia (*n* = 6), and diabetes (n = 3) attributed their feeling of self-acceptance and positive outlook to their religious faith. One participant with leprosy said: *“I’ve accepted it from Allah. Allah probably gives me this because Allah loves me (Male, 42, I13)”.*

### Age: sympathy for young, apathy for old

Some participants also talked about how their age added on to their experience of stigma and discrimination in their lives. People with HIV talked more about the discriminatory policies in the health system which restricts people younger than 18 to access HIV testing and services without the presence of their parents (macro level). This discouraged young people to access HIV services. One participant said: *“The law and policies limit younger people’s access to HIV services. For those who are under 18 years old, they need parents’ supervision [for HIV test and treatment]. Meanwhile it’s impossible for them to open up to their parents (Female, 31, I8)”.* Participants with schizophrenia talked about how the practice of *“pasung”* (shackling/forced captivity) was more common in case of younger people as they lacked agency.

Those with diabetes discussed how the society judged older people – blaming them for bringing the disease onto themselves by reckless dietary choices over their lifespan (meso level). One participant said: *“When my friends heard I had diabetes, they were like - ‘ahh that’s common, you are over 40…. you have eaten so much, so it’s common’ (Female, 56, I29)”.* For younger people, the public perception and action were mixed and varied: people either ridiculed them, did not believe they had diabetes, felt sorry for them, and/or judged and blamed their parents for their health condition. Younger individuals, especially those born with HIV, and schizophrenia were also reported to receive concern and sympathy from others. However, in micro level, participants with schizophrenia talked about how older individuals managed their condition better because of their life experience, while younger individuals lacked experiential knowledge. However, younger people were reported to be more eager to re-integrate into the society as they were *“optimistic about their future”*, while older people mostly isolated themselves from the society.

### Co-morbidity/ multiple diseases: the more the disease, the more the stigma

Nine participants talked about how having another disease or health condition compounded on their experience of stigma of living with a health condition. Participants talked about different co-morbid conditions they had: those with HIV reported of drug addiction, those with diabetes talked about having other disorders like obesity, high blood pressure, and hypercholesterolemia, while those with schizophrenia talked about being affected by a co-morbid mental illness like bipolar disorder and depression.

Participants mostly talked about experiencing stigma in the meso and micro levels. Participants with HIV specifically talked about how the judgment and devaluation from the society is intensified when a person has both HIV and drug addiction (meso level). They are particularly judged harshly and blamed for contracting HIV because of their drug use behavior. One female participant with HIV talked about how it was even harder for her *“During my pregnancy, as a woman with HIV with history of drug usage, I faced so much judgment and backlash from the society and health providers that added to the already existing difficulties I had from history of unhealthy sexual behavior, repeated abortion, and miscarriage. Those things made the process even harder (Female, 40, I2)”.*

Participants with schizophrenia with co-morbid mental illnesses reported of intensified stigma because of their conditions (micro level). One participant talked about the vicious cycle of stigma he experienced because of his three mental illnesses - schizophrenia, bipolar disorder, and clinical depression: *“Having these different mental conditions made me often feel irritated with life […] I once kicked and broke the glass windows in the mosque and at my neighbor’s house and had to get stitches and hospitalized […] and I rarely took care of myself. My family and friends distanced themselves from me. They saw nothing good in me. This made me feel even worse (Male, 38, I28)”.*

### Disability: visible targets for stigmatization

Among participants with leprosy and schizophrenia, having a disability added onto the experience of stigma and its severity. Participants with leprosy who had disability reported of experiencing stigma more frequently as they were easily noticed and identified because of their varied physical deformities and/or impairments (meso level). One participant recounted his experience of multiple stigmas in the meso level: *“People around me saw my disability and they thought leprosy was infectious, so they didn’t want to get close. Even the health staffs sometimes felt insecure, so they didn’t want to shake hands with me”,* which negatively impacted his own self-perception and self-confidence (micro level). He continued *“Because of the impairment I got because of leprosy, I felt like I couldn’t be normal again…I did not feel right in my mind.. my condition was bad for a year. I shut myself and thought I could not live my life all by myself (Male, 42, I13)”.*

For participants with schizophrenia, their disability was reportedly not as *“obvious”*. They talked about how at a first glance people would not recognize the disability associated with their health condition unless they see someone having a manic or psychotic episode. They reported that people are usually in disbelief when they talk about their disability, often asking them *“are you serious?”* or *“is it really like that?”*. One participant recounted such a similar experience: *“It was only when they saw me having a relapse that they seem dumbfounded and believed that it [disability because of schizophrenia] was real (Male, 38, I28)”.* They then discussed how the disability associated with schizophrenia intensifies stigmatization of those affected as they are no longer able to work and contribute to the society, and are instead deemed aggressive and crazy. One participant said: *“People just simply push away people with schizophrenia, because they think that those with mental health issues have already lost their mind. That’s the stigma we face (Male, 32, I40)”.*

Participants with schizophrenia also talked about micro level experience of stigma. They urged that schizophrenia causes both mental and physical disability. One participant added: *“I think schizophrenia is both a physical and mental disability. Physically it disturbs a person’s sleep quality which leads to weak immunity and physical ability to function. Mentally, it reduces our problem-solving ability (Female, 37, I21).”* They reported that because of such dual debilitating effect of schizophrenia and the resultant experiences of stigma in the society, they feel deep shame and try to conceal their condition by isolating themselves.

### Sexuality: sin, judgment, and rejection

Three participants with HIV and one with schizophrenia talked about how their sexuality (sexual orientation/identity) added on to the stigma of having a health condition. Participants talked about how the negative portrayal of LGBT in the Indonesian society leads to their stigmatization (macro level). They talked about how gays and transgender people are considered *“sinful”* and are perceived to be involved in *“bad deeds”* ascribing it primarily to the practice of anal sex. They reported how such prevalent norms are used by societal actors to justify the social exclusion and ostracization against LGBT (meso level). One participant with HIV recounted his experience where someone told him he must have been *“punished by god for denying his sexual role as a male (Male, 43, I34).”* Further, people with HIV also talked about stigmatizing experiences in health setting, where they felt judged by health service providers. One participant said: *“The health counselor started giving me inappropriate advice like – you should get a girlfriend, and why don’t you just get married? That made me feel so uncomfortable. As far as I know, a counselor should not be giving such suggestions. He should only focus on providing health-related service (Male, 44, I36).”* Two participants with HIV - one who identified as gay and other as transgender - also reported of being involved in sex work, which further fueled their negative experiences of stigma. They talked about how they were viewed as *“dirty”* in the society and discussed a multitude of negative experiences - from abuse they faced from the police and government officials to discrimination they had to encounter in the health setting because of being a sexual minority with HIV involved in sex work.

One participant with schizophrenia who identified himself as gay talked about how having the disease and also being gay has created multiple issues in his life (micro level). He said: *“I already feel the stigma of having schizophrenia. But because I am gay – there is another stigma that I face. I not only get judged because of my disease, but I am also taunted and ridiculed by my neighbors for being gay and effeminate. Its incriminating. Also, I have added stress because I can’t find a partner for life. It’s making my life hard (Male, 37, I37).”*

#### Intersectionality of multiple adversities with health-related stigma

Participants described experiencing adversities from multiple social inequalities and/or identities they hold, which intersected with the stigma related to their health condition. Table 3 presents four case studies illustrating this intersection and interplay of health-related stigma and adversities from different inequalities/oppressive identities. The case studies attempt to show a bigger picture of how the intersection of multiple adversities and oppressions manifest in an individual’s life by highlighting the personal experiences of four individuals with HIV, leprosy, schizophrenia, and diabetes.

**Table 3:** Case studies highlighting intersectional experiences of multiple adversities including health-related stigma

Case study 1: ***HIV, sexuality, comorbidity, poverty & religion***	Case study 2: ***Leprosy, disability, poverty & religious faith***	Case study 3: ***Schizophrenia, poverty, sexuality & religion***	Case study 4: ***Diabetes, comorbidity, gender, poverty & religion***
Fadi^a^ is 44 years old and lives in Jakarta. He identifies as a gay man and has been living with HIV for 6 years. He describes how poverty forced him into working as an exotic dancer and occasionally as a sex worker to make ends meet. He also got addicted to using *shabu* (methamphetamine). He knew of his HIV status in the prison when he was incarcerated for engaging in sex work. He talks about how being a gay sex worker with history of drug use and having HIV subjected him to harsh judgment and treatment from people in the community and healthcare providers. He talks about how health staffs stigmatized and discriminated against him particularly for being gay with HIV. He talks about one particular incident where he fell very sick but the hospital officials claimed he couldn’t be hospitalized *“They said I couldn’t stay there because they were afraid the disease could spread easily to other patients. They did not know well. They feared HIV, but also homosexuality”.* Further, being Muslim even worsened the stigma he felt because of his health condition. He talks of how his religious affiliation added on to the experience of stigma of HIV: *“In my religion, there is still antipathy [towards people with HIV]. It is even worse when you are gay. Religion is hard to be mixed with the health issue and even harder when it comes to your sexuality [being gay]”.*	Ali^a^ is 42 years old and hails from Cirebon. He has been living with leprosy-related disability for the past 8 years. He talks about how he used to work in Jakarta as a street vendor when he started getting blisters and wounds on his skin, which was later confirmed as leprosy. He was *“too ashamed”* to seek treatment at first. However, by the time he sought treatment - it was too late and he got impairment on his feet and hands. He talks about how the public perception of leprosy being infectious was even more intensified when people saw his impairment. He says: *“people with leprosy are ostracized and excluded in my society. So, the stigma is high. But for those who have impairment like I do, life is just impossible”*. He recounts his experience of how people would avoid him and say *“do not touch him”.* He describes how even healthcare providers showed disgust towards his impairment and avoided physical contact. Further, being poor worsened his experience of stigma in the community. He talks about how being poor affected his self-reliance and confidence. He says: *“I wanted to rise up – but I could not because of my economic condition”*. Luckily, his faith in god led him to accept his disease, and in conjunction with continued treatment - he gained back his confidence and was able to overcome and dismiss the rejection and judgment from others.	Fauzan^a^ is a 37-year-old man from East Jakarta. He identifies as a gay man and belongs to a poor devout Muslim family. He was diagnosed with schizophrenia in 2005. After being diagnosed, his family thought his mental illness was because of his “sin” and instead of seeking medical care, forced him to repent and remember *Allah*. Eventually after all attempts failed, he was sent to a mental asylum which almost bankrupted his family. He recounts how poverty worsened the stigma of his mental illness: *“The hardest thing in my life was my financial condition. My family was in deep debt. I did not have money to buy medicines. I did not have money to eat. I was afraid of losing my life. I was always worried about how I was going to survive”.* Further, the stigma of being gay even made his experience worse, which left him feeling a deep shame, sorrow, and loneliness. He says *“I experienced a lot of stigma because of my health condition, but being gay made it even unbearable. I lost all hope. I felt very lonely”.* However, he talks about how he was able overcome his adversities by having faith in god and focusing on his treatment, which made him accept himself and feel better. He says: *“I never lost my faith in god. I did not forget to pray. Besides that, the most important thing that helped were the medications. I had a holistic recovery – with faith, medicine, and self-acceptance”.*	Catherine^a^ is 52 years old and lives in West Jakarta. She says she has always been obese and when she first found out that she had diabetes 10 years back - she broke down. The added stigma of diabetes with the already existing stigma of obesity took a toll on her. She says *“I cried when I knew about it. What am I supposed to do when my body is as big as this?”*. Further, when doctors prescribed her insulin injections because of her comorbidity, it made her feel even worse. She says she thought she had a *“nasty disease”* and felt disgusted with herself. She also talked about how the society judged her harshly because she is a woman and blamed her for being *“lazy”* and making *“bad dietary choices”*. Further, she talked about how diabetes affected both her and her husband’s sexual libido. With both she and her husband living with diabetes, she talks about how their economic condition affects their life with diabetes: *“Diabetes makes us dependent on medicine, and some treatments need a great amount of money. The rich will have more options, while we just have to accept whatever we can afford”.* Despite the hardships, she highlights the importance of having faith in god: *“My life is in God’s hand. God pays attention to the most trivial things. Be grateful and accept it. Once you accept it, it will be better. It will set you free”.*

^a^ The names of the case study participants have been changed

## Discussion

This study explored the intersectionality of health-related stigma with other adversities related to the varied social inequalities and/or oppressive identities borne by individuals living with HIV, leprosy, schizophrenia, and diabetes in Indonesia. We found that intersectional adversities related to gender and socioeconomic status were more prevalent, followed by religion, age, comorbidity, disability, and sexuality. While adversities arising from religion and age were experienced in the macro and meso levels, Individuals’ religious identity and age were both found to have positive impact on their lives in the micro level. Those who self-reported of having a co-morbidity or disability, or identified as a sexual minority (gay or transgender), also reported adverse experiences that intersected with that of health-related stigma.

Our findings suggest that the intersectional experiences of health-related stigma exist across macro, meso, and micro levels. In the macro level, participants with HIV, leprosy, and schizophrenia encountered oppressive and exclusionary social norms and institutional policies that devalue and discredit people with marginalized identities. In the meso level, experiences of being stereotyped, judged, and discriminated against by societal actors (family and friends, neighbors, healthcare providers) were evident. In the micro-level, the experiences in the macro and/or meso level impacted individual’s self-confidence and self-esteem, and made them feel ashamed of their health condition and incited loneliness and hopelessness. This was consistent with the study findings by Bowleg [49] who reported that the multiple social oppressions intersect at micro level while reflecting on the macro and meso level oppressions corresponding to their identity, and ultimately shape an individual’s experience. Other studies have also established that stigma is perpetuated and reinforced in the macro (structural) and meso (interpersonal) levels leading to personal experience of stigma in the micro (intrapersonal) level, which includes enacted stigma (actual experience of discrimination and exclusion) and felt stigma (internalized shame, guilt, and loneliness, and anticipation of impending stigma experiences) [50, 51].

Gender and poverty were the most reported inequalities that intersected with the experience of health-related stigma. The oppression from gender originated from the prevailing gender norms which subjugated women, while for men any deviation from their gender roles subjected them to negative judgment. Poverty, on the other hand, casted an added layer of subjugation among those affected by their health condition and hampered their ability to access health services. These findings are supported by a large body of evidence reported from studies on HIV, leprosy and schizophrenia. Studies have found that oppression associated with gender usually intersects with health-related stigma and further worsens the experience of people living with HIV [8, 52, 53], leprosy [54, 55] and schizophrenia [56, 57]. Studies also found that negative experiences of being poor conflates with health-related stigma among individuals with stigmatized health conditions like HIV and leprosy [58–60].

Discrimination emanating from religion was the third most reported experience that was predominantly felt in the macro and meso levels. Religion, as a social phenomenon, perpetuated and reinforced stigmatization of those affected. Religious norms and standards led to devaluation of individuals with health conditions, which were particularly worse for sexual minorities (gay and transgender individuals). Studies have reported how in Indonesia, religious superstitions attribute the cause of certain health conditions like leprosy and schizophrenia to evil spirits [36, 61, 62], while sexual minorities (gay, lesbians, transgender, etc.) are considered sinners who indulge in unnatural sexual practices and are persecuted [63, 64]. This intensifies the stigmatization of those who hold these identities.

However, in the micro level, religious identity and beliefs were found to have positive impact in their lives. Individuals’ religious identity and faith was found to have a mitigatory effect and helped them develop self-acceptance and self-confidence to fight stigma. Similar positive effects were also seen in the micro level among people living with schizophrenia in regards to age, which differed among younger and older individuals. While older individuals with schizophrenia were found to have more confidence on their experiential knowledge in managing their health condition and adapting ways to avert stigmatization, younger individuals were known to be more optimistic about the future and attempted to reintegrate into the society. This demonstrates the dynamic nature of stigma and how in some contexts it can be stigmatizing, and in some it can be mitigating and empowering [65]. This is also supported by the intersectionality theory which posits that people can experience privilege and oppression simultaneously depending on what situation or specific context they are in [20, 66]. Further, the findings on the positive impact of religion and age in the micro/individual level corroborates with Shih’s [67] work on “positive stigma” where she reported how certain intrinsic positive identities that an individual holds, can help in coping with, and mitigating stigma. Shih found that while multiple oppressive identities resulted in the experience of stigma, having multiple positive identities helped the participants cope with the experience of stigma in their daily lives, and also overcome its negative effects [67]. Future studies should focus on such positive identities and also privileges, and how they interact or intersect with intersectional stigma such that a fuller picture of an individual’s experience can be examined.

Oppressions associated with comorbidity, disability, and sexuality were also experienced in conjunction to health-related stigma by those who reported of having those inequalities/ identities. This is consistent with findings from other studies. Goodin et al. [68] used the term “intersectional health-related stigma” to describe the stigma resulting from convergence of multiple health conditions, and reported how HIV stigma intersects with stigma related to chronic pain that gave rise to depressive symptoms that were more severe than those inflicted by singular stigma. Studies have also reported on how disability intensifies the experience of health-related stigma among people with HIV [69], leprosy [70] and mental illnesses [71]. Further, studies on HIV stigma have reported on how gay and transgender individuals experienced resounding effect of stigma ranging from negative judgment to social exclusion and persecution [8, 72].

This study has certain limitations. While ethnicity-related differences have been reported to affect health of individuals in Indonesia [26, 73, 74], our study could not identify an intersection of ethnicity with health-related stigma. This may have been because our study was mostly based in Jakarta and west Java where majority of the people belong to the two main ethnicities – Java and Sunda. The interview participants hinted of how the experience of intersectional stigma may be worse for those of Aceh or Papua ethnicities. Future research on intersectionality should focus on such ethnic minorities in Indonesia. Further, owing to the complex nature and dynamics of intersectionality and how it shapes the experiences of stigma in individuals with health conditions, there were certain shortcomings in this study. First, in order to explain how the different social oppressions intersect with health-related stigma, the intersectional experiences had to be deconstructed to a singular inequality or identity. However, in reality such experiences exist in a dynamic state of intersection with not only multiple oppressions, adversities, and stigma, but also privileges and positive identities. Second, while in this study the experience of stigma and adversity from a singular inequality or identity was discussed in conjunction with health-related stigma, it is important to understand that such adversities related to the different inequalities/identities can exist on its own. More nuanced and deconstructed exploration of the different oppressions may be needed. Third, this study could not capture some important nuances related to intersectional experiences of health-related stigma, viz. (i) differences in experience between participants who have publicly disclosed and those who have not, (ii) differences among participants in regards to the different duration of living with their health condition, (iii) the changes in the intersectional experiences and dynamics over time and space. We recommend future studies to focus on these important differences among the participants, and to use a longitudinal study design to capture the changes in intersectional experiences over time. Because of the broad scope of the study to explore the intersectionality of health-related stigma and to avoid further fragmentation of the concepts, the experiences of stigma were not further categorized into different stigma types, and instead only discussed within the scope of the three socioecological levels in which they exist. There were also other methodological limitations in this study. We only piloted the interview guide with four participants prior to the actual data collection. While, the piloting was useful nonetheless in testing the applicability and comprehensiveness of the interview guide, we recommend a larger pilot group for future research for further rigor and robustness. There may also have been selection bias because of purposive sampling and recruitment of participants through the community-based organizations and peer-support groups. However, this was the most optimal and realistic way for participant recruitment in case of this study, as we did not have access to the patient community other than through these organizations/groups. Future studies can look into other sampling techniques like snowball sampling or respondent-driven sampling, which are proven effective sampling approaches to recruit hidden populations like participants living with stigmatized health conditions, and are known to minimize the selection bias [75, 76].

Through exploration and assimilation of intersectional experiences of health-related stigma across different stigmatized health conditions, this study has contributed to improve the conceptualization and understanding of health-related stigma. While there are many existing conceptual frameworks and models on health-related stigma [2, 50, 51], this study provides evidence-based substantiation and further extension to those frameworks to also include multi-level conceptualization of intersection between health-related stigma and other social oppressions. The findings and the conceptualization of this study have implications for health policy, practice, and research. On the macro level, it is important for policy makers to revisit and reform the policies that instigate and reinforce stigmatization of individuals based on the inequalities/identities they hold. For example, in the policies that restrict adolescents to access sexual and reproductive health services should be changed and replaced with inclusive policies that facilitate their easy access to such services. Similarly, the policies and practices of criminalization and/or persecution of sexual minorities need to be replaced with progressive measures that ensure equal rights, treatment and dignity. On the meso level, program managers can focus on reducing social stigma by combating misinformation through health promotion and awareness and challenging negative stereotypes associated with the marginalized identities. On the micro level, it is important for healthcare providers to understand and acknowledge the existence of multiple stigmas faced by persons living with stigmatized health conditions who also bear other social inequalities/oppressive identities, and implement strategies/interventions that can jointly address such intersectional stigma. The interventions may include peer-support groups, capacity building trainings, and socio-economic empowerment initiatives that focus on empowering the stigmatized individuals.

Overall, a multi-level and cross-cutting initiative to address intersectional stigma may be helpful. Previous research have shown how multi-level stigma reduction interventions jointly targeting different social spheres of life, can help alleviate both social and personal stigma among people living with leprosy [77], and mental illnesses like bipolar disorder [78] and schizophrenia [79]. There are also cross-cutting interventions which have focused on different societal issues like poverty [80] and gender norms [81], that have shown prospects in reduction of health-related stigma. It may be beneficial to bring these - otherwise siloed - aspects of stigma reduction interventions together for an integrated cross-cutting, multi-level intervention to address intersectional stigma. Future studies should thus, focus on such multi-level stigma reduction interventions which addresses stigma and adversities originating from both - the health condition and the most prevalent social inequalities/oppressive identities, based on the geographical and cultural contexts of the study population.

## Conclusion

This study explored and uncovered how the experience of health-related stigma among Indonesians with HIV, leprosy, schizophrenia, and diabetes intersects with other adversities originating from various social inequalities and oppressive identities in an individual’s life. While intersectional inequities/identities negatively affect and marginalize people with stigmatized health conditions, other positive identities can also help mitigate the negative experiences and help them in improving their self-confidence and resilience. The findings highlight the importance of acknowledging and understanding the multi-dimensional aspect of lives of people living with stigmatized health conditions, and warrant integrated multi-level and cross-cutting stigma reduction interventions to address the intersectional oppressions they experience.

## Supplementary Information

**Additional file 1:** **Supplementary File 1.** Stakeholders involved in development and cross-cultural adaptation of interview guide.

**Additional file 2:** **Supplementary File 2.** Table. Categories and sub-categories based on Inductive-deductive analytical approach.

## Data Availability

The datasets used and/or analysed during the current study are available from the corresponding author on reasonable request.
