# Supplementary material for: Intersectionality and health-related stigma: insights from experiences of people living with stigmatized health conditions in Indonesia
Source: Int J Equity Health. 2020 Nov 11;19:206. doi: 10.1186/s12939-020-01318-w (PMC7661268; doi:10.1186/s12939-020-01318-w)
Supplement: Supplementary file 1 — Additional file 1: Supplementary File 1. Stakeholders involved in development and cross-cultural adaptation of interview guide. [file 12939_2020_1318_MOESM1_ESM.pdf]

Supplementary File 1

Stakeholders involved in development and cross-cultural adaptation of interview guide

**Name**	**Country of Nationality**	**Organization**	**Expertise/Representing disease group**	**Role**
**Research Stakeholders**				
Dr. Evi Sukmaningrum	Indonesia	Atma Jaya Catholic University Indonesia	Public Health Research/HIV/AIDS	Research Director
Dr. Catherine Thomas	Indonesia	Atma Jaya Catholic University Indonesia	Psychology	Researcher
Ms. Annisa Ika Putri	Indonesia	Vrije University Amsterdam	Public Health/Epidemiology	Master Student/Research associate
Ms. Altana Mikhakhanova	Russia	Vrije University Amsterdam	Medicine/Global Public Health	Master Student/Research associate
Ms. Caryn Yachinta	Indonesia	Atma Jaya Catholic University Indonesia	Psychology/Disability	Bachelor Student/Research assistant
Ms. Shella Kostan	Indonesia	Atma Jaya Catholic University Indonesia	Psychology/Disability	Bachelor Student/Research assistant
**Community Stakeholders**				
Mr. Mujib	Indonesia	Forum Komunikasi Difable Cirebon (FKDC)	Leprosy/Disability	Resource person/ NGO representative
Mr. Bagus	Indonesia	Komunitas Peduli Skizofrenia Indonesia (KPSI)	Schizophrenia	Resource person/ NGO representative
Ms. Natasya	Indonesia	Lentera Anak Pelangi (LAP)	HIV/AIDS	Resource person/ NGO representative
Mrs. Heruna	Indonesia	BINUS University	Diabetes	Resource person
