# Supplementary material for: Intersectionality and health-related stigma: insights from experiences of people living with stigmatized health conditions in Indonesia
Source: Int J Equity Health. 2020 Nov 11;19:206. doi: 10.1186/s12939-020-01318-w (PMC7661268; doi:10.1186/s12939-020-01318-w)
Supplement: Supplementary file 2 — Additional file 2: Supplementary File 2. Table. Categories and sub-categories based on Inductive-deductive analytical approach. [file 12939_2020_1318_MOESM2_ESM.pdf]

Supplementary File 2

Table. Categories and sub-categories based on Inductive-deductive analytical approach

Inductive Category	Deductive Sub-Category	Inductive Sub-Categories/Codes	Description
**Gender**	**Macro-level**	*Social and gender norms*	Prevalent social and gender-related beliefs/perceptions and practices
		Gender roles	Socially prevalent gender-related expectations
		Imbalanced power and agency	Unequal distribution of power/ subjugation based on gender
	**Meso-level**	*Discriminatory treatment*	Unjust and discriminatory treatment based on one's gender
		Violence/Human Rights Violation	Gender-based violence/abuse (physical, psychological, etc.)
	**Micro-level**	*Shame*	Feeling of shame because of one's gender/identity
		Loneliness	Feeling of loneliness brought on by one's gender/identity
		Hopelessness	Feeling of hopelessness attributed to one's gender/identity
		Biological factors	Biological differences, characteristics, and outcomes based on one's gender/sex
**Socioeconomic status**	**Macro-level**	*Social standing in the community*	Prevalent social hierarchy based on one's socioeconomic status
		BPJS - Indonesian Health Insurance	The pros/cons of the Indonesian health insurance policy (access, coverage, etc.)
	**Meso-level**	*Different treatment in society*	Unequal treatment by societal actors based on one's socioeconomic status
		Human rights violation	Abuse and violation of one's human rights based on one's social standing in the community
	**Micro-level**	*Ability to afford medicines/health services*	An individual's ability to access/afford health services and medicines
		Entrapment in Vicious cycle (of poverty)	Vicious cycle wherein the impact of poverty further worsens a person socio-economic condition, and vice versa
		Negative effect: self-acceptance	The negative effect of poverty that impedes in an individual's acceptance of self and one's health condition
**Religion**	**Macro-level**	*Religious beliefs/misconceptions*	Religious beliefs/perceptions/misconceptions/superstitions related to one's health/health condition/disease
		Religious/social norms	Prevalent religious/social/cultural norms and practices and their implication on one's health/health condition/disease
	**Meso-level**	*Religious persecution/discrimination*	Discrimination based on socially held religious beliefs
	**Micro-level**	*Negative effect: Non-disclosure*	Non-disclosure of one's health status to avoid religious stigmatization
		Positive: Personal religious identity	Personal religious identity as a source of positivity and hopefulness in one's life
		Positive effect: Self-acceptance	Positive effect of one's religious belief and faith on accepting oneself
		Positive effect: positive outlook	Positive effect of one's religious belief and faith on having a positive outlook on life
**Age**	**Macro-level**	*Policies & practices*	Discriminatory policies and practices on grounds of a person's biological age
		Social norms	Prevalent social norms/beliefs/perceptions/attitudes/practices in regards to a person's biological age
	**Meso-level**	*Societal judgment*	Judgment from the societal actors on grounds of a person's age
		Discriminatory treatment	Discrimination and discriminatory acts by societal actors towards a person because of his/her age
		Sympathy and concern for young	Feelings of sympathy, care and concern shown by societal actors for younger individuals with a health condition
		Apathy and judgment for old	Feeling of apathy and judgment shown by societal actors for older individuals with a health condition

	**Micro-level**	*Positive aspects*	Individual positive experiences on grounds of a person's age

**Co-morbidity**	**Macro-level**	*Discriminatory health policies & practices*	Health policies & practices that intentionally or unintentionally discriminate against individuals with co-morbidity
	**Meso-level**	*Intensified judgment*	Negative judgment from societal actors on grounds of a person's multiple health condition
	**Micro-level**	*Intensified and layered self-stigma*	Personal/self-stigma because of the existence of more than one health condition

**Disability**	**Macro-level**	*Social stereotypes and labelling*	Prevalent social stereotypes and labels regarding any form of disability
	**Meso-level**	*Easy targets for stigmatization*	Visual targeting/identification of people with disabilities by societal actors
		Discrimination	Discrimination from societal actors because of one's disability
		Misconception	Societal actor's misconception regarding different disabilities
		Negative Judgment	Negative judgment from societal actors on grounds of a person's disability
	**Micro-level**	*Effect in Job opportunities*	Personal experience of hardships in employment/job seeking/job retention
		Concealment	Concealment of one's disability/health status to avoid negative judgment/discrimination
		Shame	Feeling of shame for having a disability
		Self-isolation	Act of isolating oneself from others/society on grounds of one's disability

**Sexuality**	**Macro-level**	*Societal norms/stereotypes*	Prevalent social stereotypes and labels regarding any form of sexuality/sexual orientation
	**Meso-level**	*Social exclusion*	Act of excluding an individual from social activities based on his/her sexuality/sexual orientation
		Ostracization	Ridicule/negative judgment/discrimination based on one's sexuality/sexual orientation
	**Micro-level**	*Multitude personal issues*	A complex set of experiences of personal issues/adversities based on one's sexuality/sexual orientation
